# Structural racism as a leading cause of racial disparities in breast cancer quality of care outcomes: a systematic review

**DOI:** 10.3389/fonc.2025.1562672

**Published:** 2025-07-16

**Authors:** Ola Abdelhadi, Michelle Williams, Alice Yan

**Affiliations:** Stanford Healthcare, Stanford, CA, United States

**Keywords:** breast cancer, structural racism, SDOH, quality of care, cancer care

## Abstract

**Background:**

Non-Hispanic Black women have a disproportionately higher breast cancer mortality rate compared to non-Hispanic white women. Structural racism embedded within societal systems plays a fundamental role in perpetuating these persistent disparities. This systematic review aims to examine the relationship between structural racism and breast cancer quality of care outcomes across various racial and ethnic groups.

**Methods:**

Following the PRISMA guidelines, we conducted a systematic review of PubMed, Embase, and CINAHL for studies published until October 30, 2024, that examined the relationship between structural racism and breast cancer quality of care outcomes. We employed the Healthy People’s Social Determinants of Health (SDOH) framework to identify structural racism measures within these five themes: economic stability, education access, healthcare access, neighborhood and built environment, and social and community welfare. Breast cancer quality of care outcomes were assessed using the Donabedian quality of care model which encompasses three components of quality: process measures, structural measures, and outcome measures.

**Results:**

We conducted a systematic review of 262 studies that included at least one measure of structural racism linked to a breast cancer quality of care outcome. Of these, 29 studies met the eligibility criteria for inclusion. The most frequently examined measures of structural racism were those related to residential segregation and redlining, which pertain to neighborhood and built environment SDOH domains. The predominant finding across the studies was that both residential segregation and redlining were significantly associated with adverse breast cancer outcomes. Theses outcomes included higher mortality rates, later-stage diagnoses, and suboptimal treatment. These effects exhibited variability based on race, comorbidity, and neighborhood characteristics, highlighting the complex role of structural racism in perpetuating disparities in breast cancer outcomes.

**Conclusion:**

The complex relationship between measures of structural racism and breast cancer quality of care outcomes underscores the necessity for ongoing research to understand the pathways through which structural racism impacts health outcomes. Understanding these pathways is essential for developing targeted interventions and promoting health equity in breast cancer care.

## Introduction

Breast cancer is the most common cancer diagnosis in the United States. Yet, significant disparities in survival rates persist, particularly between non-Hispanic Black and non-Hispanic White women. Non-Hispanic Black women face a 41% higher mortality rate compared to non-Hispanic white women (1). Structural racism is a likely contributor to these disparities, as it creates adverse upstream determinants of health. While there is growing recognition of the role of structural racism in health disparities, the current body of research on its relationship to breast cancer quality outcomes remains limited. Existing studies often fail to comprehensively examine the multiple dimensions of structural racism and their impact on different breast cancer quality outcomes. The objective of this study is to examine how structural racism affects disparities in quality-of-care outcomes for breast cancer among various racial and ethnic groups.

Structural racism is defined as macro-level systems, policies, and practices that institute racial discrimination in domains such as housing, education, employment, earnings, benefits, credit, healthcare, and criminal justice (2). While systemic racism refers to the racial inequalities within systems and policies, structural racism emphasizes the interconnectedness of these systems, which reinforce inequalities over time. These interlocking systems create cumulative disadvantages for racial and ethnic groups, significantly shaping the experiences and opportunities available to breast cancer survivors from marginalized backgrounds (3).

Structural racism operates through societal power dynamics-economic, political, and social-that influence social determinants of health (SDOH), such as economic stability, education access, social context, healthcare access, and neighborhood environment (4). The Healthy People framework recognizes these factors as critical to health, functioning, and overall quality of life (5). Discriminatory practices within these domains, including redlining in housing, funding disparities in education, biased hiring, wage gaps, and healthcare access barriers, collectively impact marginalized communities (8). For example, redlining and unequal housing opportunities lead to segregated and impoverished neighborhoods, predominantly affecting specific racial and ethnic communities. In education, funding disparities and unequal access to quality schools result in differential educational outcomes across racial groups. In the economic domain, employment is similarly impacted through biased hiring practices, wage gaps, and limited career advancement opportunities for racial and ethnic minorities. In the social domain, the criminal justice system contributes to disproportionate rates of incarceration among specific communities through racial profiling, biased policing, and sentencing disparities. Similarly, in healthcare access domain, structural racism is evident in barriers to access, discriminatory practices, and health outcome disparities (9).

Previous studies have highlighted structural racism as a fundamental driver of health disparities among breast cancer survivors by influencing the broader landscape of SDOH (6). Individuals from marginalized racial and ethnic backgrounds often face multiple barriers to timely, high-quality healthcare (7), including disparities in early detection, diagnosis, and treatment, which leads to delayed interventions and worse outcomes (8). Structural racism also leads to unequal socioeconomic opportunities, further affecting access to supportive care and post-treatment services (9, 10). The cumulative effects of discrimination, stress, and limited access to healthcare contribute to higher levels of psychological distress among minority breast cancer survivors (11, 12). These findings underscore the urgent need to examine how structural racism influences breast cancer care outcomes and to implement targeted policy interventions that address the root causes of these disparities (13).

This review aims to map existing evidence on structural racism’s role in breast cancer disparities and outline areas for future research to inform policies aimed at mitigating these disparities. By examining a wide range of structural factors guided by the Healthy People 2030 SDOH framework, we seek to capture the complex interplay between various determinants of health. This holistic approach allows us to identify patterns and associations that may not be evident when focusing on isolated factors. Our study highlights areas where research is lacking. directing future research efforts toward understanding the nuanced effects of structural racism on breast cancer outcomes. By addressing structural racism and its impact on social determinants of health, we can better understand the pathways through which it contributes to adverse outcomes for breast cancer survivors from marginalized groups and develop strategies to improve equity in cancer care.

## Methods

### Data source and search strategy

In adhering to the PRISMA (Preferred Reporting Items for Systematic Reviews and Meta-Analysis) guidelines (50), we searched three databases: PubMed, Embase, and CINAHL for English-language, peer-reviewed studies on structural racism and breast cancer outcomes till October 2024. Guided by the Healthy people’s 2030 SDOH framework, we searched for studies that measured and examined structural racism, which includes social and community context, economic stability, education access, healthcare access, neighborhood and built environment domains.

These aspects were considered in relation to quality-of-care outcome measures, process measures and structural measures. These measures include mortality, survival rates, treatment, biomarkers, and incidence rates, screening rates, as well as other quality of care measures We identified specific Medical Subject Heading (MeSH) terms and keywords that encapsulated the concept of structural racism and structural inequalities related to breast cancer outcomes.

### Study selection: inclusion and exclusion criteria

Studies met inclusion criteria if they (1): published in English, (2) had a data sources within the United States, (3) fall into one of the following designs: cross-sectional, cohort, randomized clinical trial (RCT), quasi-experimental, or pre-post study design, (4) consist of adults aged 18 years or older, (5) had to encompass a racial or ethnic minority group or multiple racial and ethnic groups, (6) measure at least one breast cancer outcome such as a) outcome measures b) process measures, or c) structural measures, (7) measure one of the structural factors, as defined by the Healthy People’s SDOH framework, including a) Economic, b) Social, c) Healthcare access, d) build environment, or e)education.

We excluded studies that (1) examined racism only at the individual level and did not examine structural measures including systems, policies, and practices, (2) Studies with Abstract only, (3) Qualitative and review studies.

### Data extraction and assessment of bias

**Figure 1:**
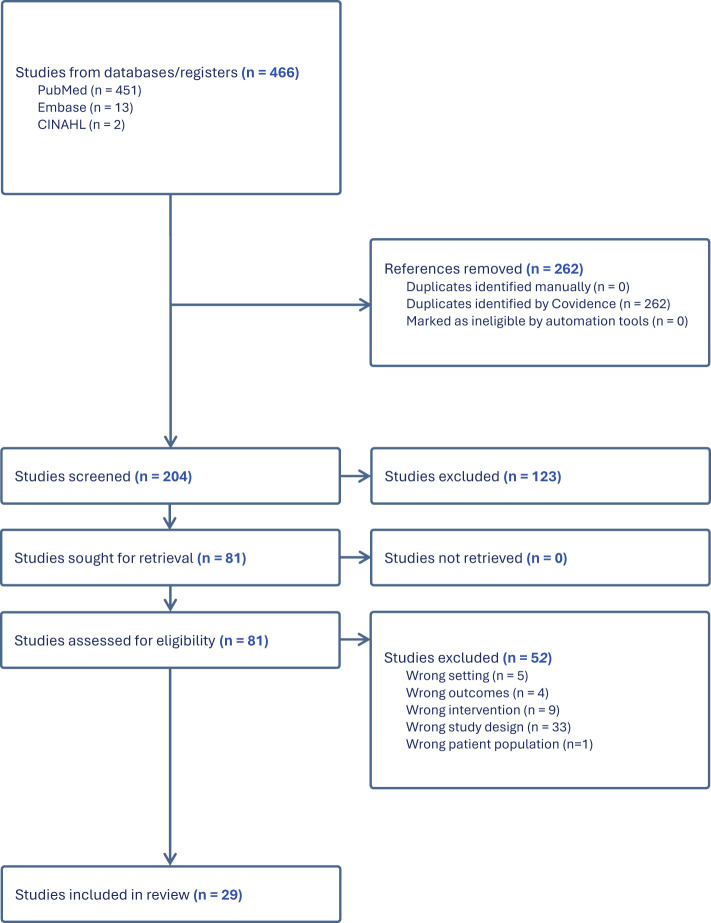
PRISMA flow chart for study selection.

Two independent reviewers (OA and AY) screened all the results to determine eligibility for this review. Studies were evaluated for bias and quality using the Joanna Briggs Institute (JBI) Checklist. **Figure 1** provides an overview of the study selection process. We began by reviewing the titles and abstracts of potential studies to examine their alignment with our inclusion criteria. To maintain consistency, a checklist was utilized, encompassing our eligibility criteria, to make the determination of whether a study should be included or excluded. Any studies that did not meet the eligibility criteria were excluded from further consideration. Following this initial assessment of titles and abstracts, full-text articles that met our initial inclusion criteria proceeded to a more comprehensive synthesis. Both the initial and full-text reviews were conducted independently and then collaboratively finalized by all authors, using the platform Covidence (14). This collaborative use of the checklist ensured uniform decision-making processes for each article under review. Upon the completion of full-text synthesis, articles that did not meet the inclusion criteria were excluded, and the rationale for their exclusion was documented. During the data extraction phase, we captured critical information, including study design, study population, sample size, and the specific outcomes assessed.

### Search, study selection, and data collection

**Figure 1** shows the PRISMA diagram with the results for study identification, screening, eligibility, and final selection, along with details of studies excluded and retained at each phase. After searching PubMed, Embase, and CINAHL, 466 studies were identified. The search strategies are included in **Table 1A** in the Appendix. Following the removal of duplicates, 262 articles remained for title and abstract screening, using the inclusion criteria listed above.

There were eighty-one articles that met the inclusion criteria and underwent a full article review. Of the eighty-one articles that initially met our inclusion criteria, 52 were subsequently excluded due to reasons such as inappropriate study design. This led to a final inclusion of 29 articles for our synthesis. The articles included for data extraction are shown in **Table 1**.

**Table 1:** Study design, structural racism measures, and outcome measures.

First author, year	Study design and data	Population and sample size	Measure of structural racism	Breast cancer outcomes
Beyer, 2016 (15)	Retrospective cohort, Milwaukee, Wisconsin cancer registry	Black/African American women diagnosed with breast cancer (n=1010)	Racial bias in mortgage lending and redlining using redlining index	Breast cancer survival among Black/African American women
Beyer, 2021 (16)	Retrospective cohort, SEER	Non-Hispanicwhite, Hispanic, and non-Hispanic Black women aged 66–90 years with an initial diagnosis of stage I-IV breast cancer (n=27,516)	Redlining index	Survival among older women with breast cancer
Bikomeye, 2023 (17)	Retrospective cohort, SEER	Women(n=18,119)	HOLC risk grade	receipt of various cancer treatments, all-cause mortality (ACM), and BC-specific mortality (BCSM)
Bonner, 2019 (36)	Cross sectional, population-based sample	Black, Hispanic, and white women in Northern California with stage I to III breast cancer diagnosed (n=500)	Location quotient (LQ) of residential segregation	receiving guideline-recommended adjuvant therapy and patient knowledge of tumor characteristics
Canales, 2023 (18)	Retrospective cohort, SEER	Non-Hispanic Black women aged 66–90 years with an initial diagnosis of stage I-IV BC (n=5,231)	Local LEx/Is and MSA measures of isolation	Survival among older non-Hispanic (NH) Black women with breast cancer (BC)
Collin, 2021 (19)	Retrospective cohort, Georgia Cancer Registry data	Non-Hispanic white (n=4,943) and Non-Hispanic Black (n=3,580) women with a first primary invasive breast cancer	Redlining index	breast cancer mortality
Dai, 2010 (37)	Cross sectional, Michigan Cancer Surveillance Program.	Women consisting of 68.9% whites, 25% Blacks, and 6.1% of other minorities (n=4,043,467)	Isolation index	Late-stage diagnosis of breast cancer
Eldridge, 2022 (20)	Retrospective cohort, SEER, 12 states represented in the data set were California, Connecticut, Georgia, Hawaii, Iowa, Kentucky, Louisiana, Michigan, New Mexico, New Jersey, Washington, and Utah.	Non-Hispanic white women (n=301,600) and non-Hispanic Black women (n=46,853)	Black to white rate ratios in educational attainment, political participation, incarceration, and unemployment; and dichotomized to “high” or “low” structural racism using the median rate ratio of the 12 states	Incidence of triple-negative breast cancer (TNBC)
Goel, 2022 (21)	Retrospective cohort, Local Cancer registry in south Florida	Non-Hispanic Black and Non-Hispanic white women with breast cancer(N=5909)	Index of concentration at extreme (ICE)	Breast Cancer Specific Survival
Haas, 2008 (38)	Cross sectional, Surveillance, Epidemiology, and End Results (SEER)	Black and white women aged 66 to 85 years diagnosed with stage I, II, or IIIA breast cancer (n= 70,541)	Isolation index	Receipt of adequate breast cancer care
Krieger, 2016 (39)	Cross sectional, US Surveillance, Epidemiology, and End Results (SEER) program.	Black and white women aged 25–84 who were diagnosed with primary invasive breast cancer (n= 516,382)	Index of Concentration at the Extremes (ICE).	ER+ve breast cancer
Krieger, 2020 (22)	Retrospective cohort, Massachusetts cancer registry	Non-Hispanic white and Non-Hispanic Black breast cancer women (n= 20,808)	HOLC risk grade	Late stage at presentation
Lubarsky, 2024 (31)	Retrospective cohort, Two medical centers in Miami	(n=5173)	Index of concentration at Extreme	Receipt of receipt of National Cancer Center Network (NCCN) guideline-concordant breast cancer treatment
Michaels, 2022 (34)	Retrospective cohort, California Cancer Registry	Women with primary invasive breast cancer (n=118,381)	Racial bias in mortgage lending	Incidence of luminal A and TNBC
Miller-Kleinhenz, 2024 (43)	Cohort, Georgia Cancer registry	Women diagnosed with breast cancer (n=1764)	Redlining index, persistent mortgage discrimination using both contemporary mortgage discrimination and redlining scores	ER status, late stage at diagnosis, BC-specific death
Miller-Kleinhenz, 2023 (23)	Retrospective cohort, Georgia Cancer registry, 80 NHB and NHW women diagnosed with stage I to III breast cancer	Non-Hispanic white (n=17) and Non-Hispanic Black (n=63) women diagnosed with BC	Redlining index	DNA methylation in breast tumor tissue
Moss, 2019 (40)	Cross sectional, National Cancer Institute’s Health Information National Survey	Non-Hispanic white and other races (n=17 736)	Dissimilarity index (DI)	Breast cancer screening
Nabi, 2024 (33)	Retrospective cohort, SEER	Non-Hispanic Black and white women with Ductal carcinoma *in situ* (DCIS) (n=103,898)	Index of concentration at Extreme	Mortality and mastectomy and radiotherapy
Ojinnaka, 2017 (24)	Retrospective study, Texas Cancer Registry	Black, white, Hispanic and non-Hispanic women with breast cancer(n=69,824)	Isolation index	Using Mastectomy/Breast Conservation Therapy
Oka, 2022 (41)	Cross sectional, Tennessee Cancer Registry (TCR)	Non-Hispanic white (n=46,983) Non-Hispanic Black (n=7,967) diagnosed with a non-invasive or invasive breast cancer	Isolation index of P*	Breast cancer condition (invasive/non-invasive)
Pittell, 2024 (32)	Retrospective cohort, Flatiron Health electronic health record (EHR)	Women with metastatic breast cancer (n=27,459)	Index of concentration at Extreme Secondary: Yost Index % of Black Structural Racism Indicator	Overall survival and time to treatment initiation
Plascak, 2021 (42)	Prospective Cohort, questionnaire to patients from New Jersey State Cancer Registry	Black breast cancer survivors (n = 476)	Gini and Isolation indices	Breast cancer reported levels of stress
Plascak, 2022 (25)	Retrospective cohort study, New Jersey State Cancer Registry women with breast cancer	Latina (n=2869) Non-Latina Black (n=3506) Non-Latina white (n=7686) Other (n=1083) women with breast cancer	HOLC risk grade	Late stage at diagnosis, high tumor grade, triple-negative subtype (lacking estrogen receptor, progesterone receptor, and human epidermal growth factor receptor 2 expression), breast cancer–specific death.
Poulson, 2021 (26)	retrospective cohort study, SEER	Black and white patients diagnosed with invasive breast cancer, 100 most populous participating counties	The racial index of dissimilarity	advanced stage at diagnosis (stage III/IV), surgery for localized disease (stage I/II), and overall stage-specific survival
Pruitt, 2015 (27)	Retrospective cohort, Texas cancer registry	Black, Hispanic and white women (n=109,749)	Location quotient of residential racial segregation (LQ) measure	mortality among breast cancer patients
Reeder-Hayes, 2024 (35)	Retrospective cohort, North Carolina Central Cancer Registry	Women with stage I-III BC (n=32,095)	Structural racism composite score from eight SDOH across five domains	Treatment delay
Russell, 2012 (28)	Retrospective cohort, Georgia Comprehensive Cancer Registry (GCCR)	Black and white women diagnosed with breast cancer (*n* = 22,088)	Theil’s Information Theory Index (H index)	breast cancer and all-cause mortality among Black and white breast cancer patients
Warner, 2010 (29)	Retrospective cohort, California Cancer Registry (CCR),	Black (n=10,030) and white (n=113,979) women in California	Dissimilarity, delta, isolation, relative centralization, and spatial proximity indices.	all-cause and breast cancer-specific survival between and within Black and white women diagnosed with breast cancer
Wright, 2022 (30)	Retrospective cohort, Massachusetts cancer registry	Black, Hispanic, white non-Hispanic, Asian or Pacific Islander, American Indian or Alaska Native, and other racial groups of breast cancer patients (n=60,173)	HOLC risk grade	Breast cancer Incidence

## Results

### Study design, sample characteristics, and settings

The characteristics of the twenty-nine studies included in this review are summarized in **Table 1** and **2**. Out of the 29 studies reviewed, 21 were retrospective cohort studies (15–35), while the remaining study designs included 6 cross-sectional studies (36–41) and 2 prospective cohort study (42, 43). The samples comprised adult women aged >18 years, diagnosed with breast cancer, and representing at least one racial minority group.

In terms of settings, most of the studies utilized secondary data from national and state cancer registries, such as the US Surveillance, Epidemiology, and End Results (SEER) program, as well as cancer registries in Massachusetts, California, Georgia, Tennessee, Michigan, South Florida, New Jersey, Milwaukee Wisconsin, North Carolina, Miami, Florida, and Texas (**Table 2**).

**Table 2:** Study years, geographical area, neighborhood definition and measure of association.

First author, year	Years of the Data	Geographical Area	Neighborhood definition
Beyer, 2016 (15)	2002-2011	Southeastern Wisconsin, including the metropolitan areas of Milwaukee and Racine	ZIP code tabulation area (ZCTA) average values.
Beyer, 2021 (16)	2007- 2013	United States	Census tract level within metropolitan statistical areas​​.
Bikomeye, 2023 (17)	2010-2017	United States	Census tract level within metropolitan statistical areas​​.
Bonner, 2019 (36)	2010-2011	Northern California	Census tract with the larger metropolitan statistical area.
Canales, 2023 (18)	2007-2013	United States	Census tracts
Collin, 2021 (19)	2010 - 2014	Metropolitan Atlanta area	Census tracts within the Metropolitan Atlanta area.
Dai, 2010 (37)	1998-2002	Metropolitan Detroit, encompassing Wayne, Oakland, and Macomb counties	ZIP code areas within the tri-county region
Eldridge, 2022 (20)	2010-2016	12 states in the United States	State-level.
Goel, 2022 (21)	2005-2007	South Florida	Census tract
Haas, 2008 (38)	1992-2002	United States	Census tracts and counties
Krieger, 2016 (39)	1992-2012	United States	County-level data
Krieger, 2020 (22)	2001-2015	28 municipalities in Massachusetts with digitized HOLC maps	Census tracts within these municipalities.
Lubarsky, 2024 (31)	2005-2017	Miami	Census tract
Michaels, 2022 (34)	2006-2015	California	Census tract
Miller-Kleinhenz, 2024 (43)	2010-2019	Georgia	Census tract
Miller-Kleinhenz, 2023 (23)	None specified	Not specific	Census tracts
Moss, 2019 (40)	2011-2017	United States	Census tracts within counties
Nabi, 2024 (33)	1990-2015	United States	County -level
Ojinnaka, 2017 (24)	1995-2012	Texas	Census tracts
Oka, 2022 (41)	2005-2014	Tennessee	County level
Pittell, 2024 (32)	2011-2022	United States	Census tract
Plascak, 2021 (42)	2010	10 counties in New Jersey	County level
Plascak, 2022 (25)	2008-2017	New Jersey	Census tracts
Poulson, 2021 (26)	2005 – 2015	Across the USA	County level​​.
Pruitt, 2015 (27)	1995-2009	Urban areas in Texas	Metropolitan statistical areas (MSAs), using year 2003 MSA definitions​
Reeder-Hayes, 2024 (35)	2004-2017	North Carolina	County level
Russell, 2012 (28)	1999-2003	Georgia	Census Tract; MSA/MiSA
Warner, 2010 (29)	1996-2004	California	US Census Bureau
Wright, 2022 (30)	2005-2015	28 municipalities in Massachusetts	Census tract

### Exposure and outcome measures

#### Exposure measures: structural racism measures

**Figure 2:**
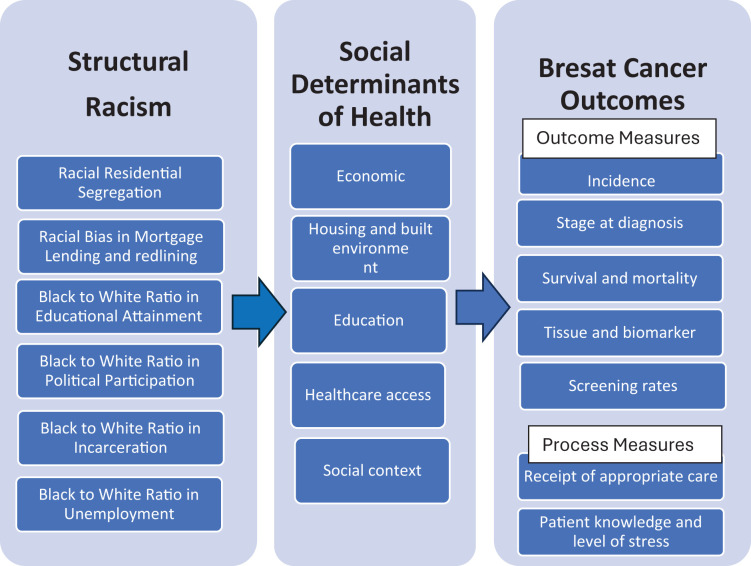
Structural racism impact breast cancer quality of care through Influence on SDOH. Structural racism as fundamental mediator for impact of SDOH on breast cancer outcomes.

Based on Healthy people framework’s social determinates of health, the neighborhood and build environment domain has been the focus of most studies examining the impact of structural racism on breast cancer outcomes. Specifically, fourteen studies investigated residential segregation, and eight studies examined redlining, which refers to discriminatory mortgage lending practices based on racial composition of neighborhood and financial disinvestment in predominantly Black neighborhoods (often referred to as “red zones”). In contrast, only one study examined structural racism in the domain of education, social and community context, and economic stability based on the Black-to-white ratio in educational attainment, political participation, incarceration, and unemployment. Notably, while many studies assessed healthcare access—such as insurance coverage and disparities in breast cancer outcomes—none specifically investigated the association between structural racial discrimination in healthcare access and breast cancer outcomes (**Figure 2**).

Below are specific measures of structural racism utilized in the studies:

**1- Residential Segregation Measures (17 studies):**

Location Quotient: This measure compares the proportion of a specific racial group in a neighborhood to the proportion of that group in a larger area, indicating the degree of concentration or segregation. (2 studies) (27, 36) Index of Concentration at Extreme: This index quantifies the extent to which a neighborhood is dominated by a single racial group, highlighting extreme segregation. (5 studies) (21, 31–33, 39).
Isolation measures: These measures assess the degree to which members of a racial group are isolated from other groups within a neighborhood (7 studies) (18, 24, 29, 37, 38, 41, 42) Delta: This measure captures changes in segregation over time, providing insights into trends in residential patterns. (1 study) (29).
Relative Centralization: This measure evaluates the centrality of a racial group within a metropolitan area, indicating how concentrated or dispersed the group is. (1 study) (29).
Spatial Proximity: This measure assesses the physical distance between different racial groups within a neighborhood, reflecting the level of integration or segregation. (1 study) (29) Dissimilarity Index: This index measures the evenness of racial distribution across neighborhoods, indicating how evenly different racial groups are spread out. (3 studies) (26, 29, 40).
Theil’s Information Theory Index (H index): This index quantifies the level of segregation by considering the distribution of racial groups across different areas. (1 study) (28).
Gini index: A common measure of segregation that assesses the evenness of racial distribution within a population. (1 study) (42).

**2-Redlining and Mortgage Bias Measures (10 studies):**

HOLC Risk Grade (grades A, B, C, D): This grading system, developed by the Homeowners’ Loan Corporation, categorized neighborhoods based on perceived investment risk, often disadvantaging Black neighborhoods. (4 studies) (17, 22, 25, 30).
Redlining Index: This index quantifies the extent of redlining practices in specific areas, highlighting the impact of discriminatory lending on neighborhood development. (5 studies) (15, 16, 19, 23, 43).
Racial Bias in Mortgage Lending Against Black Residents Regardless of Neighborhood redlining: This measure examines discriminatory lending practices targeting Black individuals, irrespective of their neighborhood’s redlining status. (2 study) (15, 34).

**3-Black to white Ratio in Educational Attainment, Political Participation, Incarceration, and Unemployment Measure:** This measure assesses disparities in these domains by comparing the educational attainment, political participation, incarceration, and economic outcomes of Black individuals to those of white individuals within same neighborhood. Educational attainment ratio measured as the relative proportion of Black individuals to White individuals over the age of 15 who hold a bachelor’s degree or higher. Employment ration assessed as the relative state-level unemployment rate ratio between Black and White individuals. Incarceration measured as the relative proportion of Black individuals to White individuals incarcerated in jails and prisons, as well as the disenfranchisement rates of Black individuals due to felony convictions. Political participation evaluated as the relative proportion of Black individuals to White individuals aged 18 and over who were registered to vote and who participated in elections. (1 study) (20).

**4- Racial Gaps in SDOH Composite Score Measure: From eight SDOH across five domains**, this composite score ranked from 0–100 minimum-maximum scale for racial gap in SDOH factors at the county level. (1 study) (35).

#### Breast cancer quality of care outcome measures

We used the Donabedian model (2005) for quality-of-care classifications into three categories: outcome measures, process measures, and structural measures. For the quality of care outcome measures, a total of 15 studies included measures of survival and mortality (15–19, 21, 25–29, 32, 33), incidence measure is included in 2 study (30 ,34), stage at diagnosis is included in 6 studies (22, 26, 37, 41–43), tissue and biomarker measures are included in 5 studies (20, 23, 25, 39, 43), and patient-centric measures are included in two studies (36, 42). For the quality of care process measures, receipt of appropriate cancer care measures are included in 9 studies (17, 24, 26, 31–33, 35, 36, 38), and a measure of breast cancer screening is included in one study (40). None of the studies investigated quality of care structural measures.

### Study findings: evidence of impact of structural racism

Residential segregation and redlining emerged as the most frequently employed measures of structural racism, showing positive associations with multiple adverse breast cancer outcomes including high mortality rates, late stage at diagnoses, and lower quality of care.

However, the influence of structural racism varies, affected not only by individual characteristics such as race and ethnicity, comorbidity, but also by neighborhood features and their interactions.

#### Residential segregation

Studies focusing on residential segregation and mortality rates have consistently found a significant impact of residential segregation on mortality.

##### Correlation with mortality rates varied based on neighborhood measures

Canales et al. (18) observed that women residing in areas with high local isolation faced increased mortality from breast cancer, particularly among those with two or more comorbidities HR = 1.20 (95%CI: 1.08-1.33). However, local isolation was not associated with higher mortality when it was linked with a high level of segregation in the Metropolitan Statistical Area (MSA). Protective ethnic density may play a role in attenuating this effect. Goel et al. (21) identified higher hazards of death in predominantly non-Hispanic Black and Hispanic segregated neighborhoods especially in low-income communities HR= 2.43 (95%CI: 1.72- 3.43) and HR= 1.99 (95%CI: 1.39- 2.84). Poulson et al. (26) noted a 29% RR=1.29 (95% CI: 1.04- 1.60) increased hazard of death with rising Index of Dissimilarity (IoD) for Black patients over 50, while white patients showed no significant difference. Pruitt et al. (27) found associations between elevated Black segregation and higher all-cause mortality HR= 1.31 (95% CI: 1.26-1.37), but the association disappeared after controlling for race and ethnicity. Russell et al. (28) highlighted the significant impact of tract-level percent Black on breast cancer-specific mortality and observed an interaction between race and MSA/MiSA area segregation. Among Black but not white, as the segregation increase the mortality increased HR = 2.20 (95% CI: 1.09- 4.45) On the other hand, Warner and Gomez (29) found that living in neighborhoods with a higher proportion of Black residents was associated with lower all-cause and breast cancer-specific mortality among Black women HR = 0.86 (95% CI: 0.76–0.97) but higher mortality among white women HR = 1.07 (95% CI: 1.02–1.13). Hass et al. (38) found that although Blacks experienced greater breast cancer mortality than whites, segregation did not substantially mediate this disparity.

##### Correlation with late stage at diagnosis

Dai (37) identified a positive correlation between Black residential segregation and the late-stage breast cancer presentation, even after accounting for primary care access, mammography access, economic advantages, and sociocultural barriers mean=0.109 (p<0.01). Poulson et al. (26) revealed that increasing segregation is associated with a higher risk of advanced-stage presentation in Black patients aged over 50 RR= 1.49 (95%CI: 1.27- 1.74). Warner and Gomez (29) noted that neighborhood racial composition and metropolitan segregation did not explain differences in cancer stage or survival between Black and white women. Additionally, Oka et al. (41) found no association between county-level Black isolation and invasive breast cancer.

##### Correlation with receiving treatment

Haas et al. (38) revealed that increased Black segregation was associated with a reduced likelihood of both Black and white women receiving adequate breast cancer care OR=0.73 (95% CI: 0.64-0.82), with 8.9% explaining a notable portion of the Black-white disparity. Hispanic-white disparities initially observed disappeared when accounting for residential segregation. Ojinnaka et al. (24) found that higher racial residential segregation decreased the likelihood of receiving mastectomy/breast-conserving treatment, particularly affecting African American individuals OR=0.56 (95% CI: 0.36-0.88). While Nabi et al. (33) found that women with DCIS in less privileged counties measured by ICE higher odds of mastectomy *vs* radiotherapy and breast conservative surgery OR = 1.51; (95%CI: 1.35–1.69) (33). Polsun et al. (51) reported a 3% lower likelihood of surgical resection for localized disease with a rising Index of Dissimilarity (IoD). In contrast, Bonner et al. (36) found a higher likelihood of receiving adjuvant hormonal therapy in participants with higher residential segregation measured by the Black Location Quotient (LQ) OR= 4.06 (95% CI: 1.26-12.93), emphasizing the nuanced and multifaceted nature of the relationship between residential segregation and breast cancer care outcomes. Lubarsky (31), found that non-Hispanic Black less likely to receive guidelines breast cancer treatment compared to non-Hispanic whites regardless the status of residential segregation (31). Pittel et al. (32) found that, compared to women in more privileged women form less privileged neighborhoods measured as more segregated had longer time to treat (38 *vs* 31 days) and less survival rate HR=0.91, (95% CI 0.86- 0.95) (31).

##### Correlation with screening rates

For breast cancer screening and segregation, Moss et al. (40) revealed that breast cancer screening rates were lower in rural compared to urban areas. This difference was not linked to segregation among breast cancer patients.

##### Correlation with perceived stress

Regarding perceived stress and segregation, Plascak et al. (42) found weak positive correlations with Black segregation (Gini index), body mass index, and perceived stress.

##### Correlation with biomarkers

Similar to redlining, studies have found that residential segregation impacts the biological and tissue markers of breast cancer characteristics. Krieger et al. (39) found higher odds ratios for ER+ versus ER− tumors in women living in the top versus bottom quintile of counties, with adjustments for income and ICE measures attenuating racial/ethnic odds ratios for being ER+.

#### Redlining and mortgage bias

The correlation between redlining, mortgage bias, and increased mortality rates in breast cancer outcomes exhibited variations based on race/ethnicity and comorbidity.

##### Correlation with mortality rates vary based on race

Beyer et al. (15) noted that while mortgage lending bias had an increase in mortality hazard rates for all cause HR=1.16 (1.04-1.29) and for breast cancer specific HR=1.12 (95%CI: 0.98-1.28), the redlining index reduced these rates HR=0.73 (0.59-0.90) for all cause morality and HR=0.76 (95%CI: 0.59 to 0.98) for breast cancer-specific mortality for Black women. Collin et al. (19) found that increased redlining metrics were associated with higher estimated breast cancer mortality rates, and these outcomes varied among racial/ethnic groups. Notably, they observed a significant positive association HR= 1.39 (95% CI: 1.09-1.78) in Non-Hispanic white women, while there was no significant association HR = 1.13 (95% CI: 0.90-1.42) detected in Non-Hispanic Black women.

Correlation with Mortality Rates vary based on Comorbidities: Beyer et al. (16) found a significant increase in mortality for non-Hispanic Black race in redlined zones compared to non-Hispanic whites HR =1.25 (95%CI: 1.08-1.46). While the hazard rate was lower for women with comorbidity in the relined zones for all races HR=0.82 (95%CI: 0.69 to 0.98). Additionally, Bikomeye et al. (17) demonstrated that historical redlining is a significant predictor of poorer survival after breast cancer diagnosis, affecting all-cause HR= 1.09 (95% CI: 1.03 - 1.15) and breast cancer–specific mortality likelihood HR= 1.26 (95% CI: 1.13 - 1.41). This relation was mediated by comorbidities as a significant contributor for mortality hazard for all races. HR= 1.16 (95%CI 1.09 - 1.23) with no comorbidities versus HR=1.09 (95% CI: 1.03 - 1.15) after adjusting for comorbidities.

##### Correlation with late stage at diagnosis

Recent research by Krieger et al. (22) also reveals elevated risks of late-stage cancer diagnosis among women with breast cancer residing in redlined areas risk ratio (RR)= 1.07 (95%CI: 0.98- 1.17). In-depth analyses of the residual disparity indicate that, even following a hypothetical intervention aimed at equalizing the distribution of census tract segregation (CT ICE), disparities in late-stage cancer risk associated with redlining, as measured by HOLC area, would persist RR=1.03 (95% CI: 0.95- 1.12).

Plascak et al. (25) discovered race and ethnicity-dependent variations in breast cancer outcomes associated with HOLC grades. Living in areas designated as “best” by the HOLC was linked to reduced chances of being diagnosed at a late stage OR=0.34 (95% CI: 0.22-0.53).

##### Correlation with receiving treatment

Bikomeye et al. (17) found that historical redlining was correlated with a decreased probability of undergoing surgery OR = 0.74 (95% CI: 0.66 - 0.83) and an increased probability of receiving palliative care OR = 1.41: (95% CI= 1.04 - 1.91) for all races.

##### Correlation with biomarkers

Additionally, studies have found that redlining impacts the biological and tissue markers of breast cancer characteristics. Miller-Kleinhenz et al. (23) identified a positive correlation between redlining and methylation in breast cancer. Miller-Kleinhenz et al. (43) found that living in historically redlining areas linked to increased odds of ER-ve breast cancer among non-Hispanic Black women (odds ratio [OR], 1.62 [95% CI, 1.01-2.60]) (43).

##### Correlation with incidence varied based on associated recent segregation

Micheals et al. (34) found no association between mortgage bias and incidence of Luminal A breast cancer (IRRadj = 0.92, 95% CI = 0.85, 1.00) (34) Studies suggest that examining historical redlining in conjunction with current residential segregation measures can provide a better understanding of the impact of structural racism. For example, Wright et al. (30) found that the highest incidence occurred in areas with the best HOLC grade and privileged contemporary characteristics, particularly for ER-positive and PR-positive breast cancer, while the lowest incidence was in areas with concentrated racialized economic deprivation and no HOLC grade.

#### Black to white rate ratios in educational attainment, political participation, incarceration, and unemployment

##### Correlation with incidence varied by race

Eldridge and Berrigan (20) found that Black women residing in states with high levels of structural racism across the domains of educational attainment, judicial treatment, and political participation were more likely to be diagnosed with triple-negative breast cancer (TNBC) compared to those in states with low disparities. Interactions between indicators of structural racism and race were significant for educational attainment, employment, disenfranchisement, and voting practices, revealing that the impact of these measures on TNBC diagnosis significantly differs between Black and white women. Disparities in educational attainment were positively associated with TNBC for both groups but were significantly weaker for white women OR=1.17 (95% CI: 1.10-1.23) compared to Black women OR=1.50 (95%CI: 1.27-1.77). In contrast, the positive association between disparities in voting practices and TNBC was observed only among Black women, being null for white women.

#### Racial gaps in SDOH

##### Correlation with treatment

Reed et al., 2024 found that living in counties with high racial gaps in eight SDOH across five domains was associated with increased odds of treatment delay among Black women with breast cancer Adjusted OR=1.2 (95% CI: 1.08 - 1.45) (35).

## Discussion

This systematic review synthesizes current evidence on the impact of structural racism on disparities in quality-of-care outcomes for breast cancer. The findings highlight that residential segregation and redlining are critical contributors to disparities in survival rates among breast cancer patients, as identified in multiple studies. Structural racism also contributes to inequities across other breast cancer outcomes, including incidence rates, stage at diagnosis, tumor biomarker profiles, and patient-centered quality of care measures. The relationship between structural racism and breast cancer outcomes is often influenced by interplay of multiple factors, including sample characteristics, such as race and ethnicity, comorbidities, neighborhood attributes, and the geographic scope of measurement (e.g., local census tract versus broader metropolitan statistical areas). These interactions underscore the complex interplay of factors shaping disparities in breast cancer care.

Structural racism functions at the macrolevel, encompassing multiple systems and creating widespread disparities The World Health Organization’s (WHO) framework of social determinants of health, (See **Appendix Figure A**), highlights the impact of structural racism on these determinants, which ultimately shapes disparities in health outcomes for marginalized communities, including those affected by breast cancer. It emphasizes the multifaceted factors that influence health outcomes, including individual, social, economic, and environmental conditions. Structural racism exerts profound influence through policies and practices such as residential segregation and redlining, which systematically disadvantage certain racial groups across multiple domains of SDOH. For example, redlining, which is a macrolevel policy, has long restricted material circumstances such as economic opportunities, educational resources, and access to quality healthcare in predominantly Black neighborhoods, which in turn contributes to adverse health outcomes such as delayed detection, diagnosis, and treatment for cancer survivors. These structural inequities result in poorer prognoses for breast cancer across multiple racial groups.

Structural racism is the overarching form of racism that encompasses various lower-level forms of racism. One prominent example is systematic racism, which operates within specific systems, such as healthcare or criminal justice, and contributes to cross-system inequalities. Disparities in healthcare coverage, underfunded schools in Black neighborhoods, and the over-policing of communities of color serve as examples of systematic racism. In contrast, institutional racism is more narrowly defined, affecting individuals within specific institutions or corporate policies. Historical practices like literacy tests for voting and criminal background checks for employment disproportionately impact African Americans are examples of this form of racism and lead to compounding disparities in healthcare. Experiencing racism across these multiple levels leads to health disparities through pathways including increased exposure to environmental pollution (44), restricted healthcare access (45), targeted marketing of harmful products (e.g., tobacco) (46), and biological effects from chronic stress, such as inflammation, and accelerated cellular aging (i.e., telomere shortening) (47).

Studies revealed varying impacts of structural racism on breast cancer outcomes. For example, racial bias in mortgage lending was associated with increased all-cause mortality hazard rates for Black women (15). Structural racism also affects non-Black women, such as increased odds of TNBC among both Black and white women in high-structural racism environments, though the impact was greater for Black women (20). In some cases, structural racism profoundly affects white communities as study by Collin et al. (19) found that the increased redlining was significantly associated with breast cancer mortality among non-Hispanic white women but less so among non-Hispanic Black women.

The relation between structural racism and breast cancer outcomes often depends on interacting factors such as race, ethnicity, neighborhood composition, and segregation levels. For instance, while Hispanic-white disparities in care diminished after adjusting for residential segregation (38), other studies, like Warner and Gomez (9), found no explanatory power of neighborhood racial composition and metropolitan segregation on cancer stage or survival differences between Black and white women. This underscores the complexity of structural racism’s impact on breast cancer quality of care, requiring an understanding of how multiple moderators interact. For example, Russell et al. (28) highlighted the significant impact of the interaction between race and MSA/MiSA area segregation on breast cancer-specific mortality rates.

This study has several limitations: First, the broad scope of measures leads to a lack of depth in understanding the impact mechanisms of each measure; however, this systematic review was able at identifying evidence for relationship between structural racism and breast cancer care outcomes. Second, we did not include assessments of bias for the studies included as many of the studies did not include it. Third, while this study may not provide the best framework for clinical guidelines, it directs attention to necessary future research.

Utilizing established frameworks, such as the Donabedian model for quality of care and the Healthy People 2030 SDOH framework, provides a structured approach to our analysis. This allows us to categorize and assess the various measures systematically. Our goal is to provide a comprehensive overview that can inform future empirical research and policy interventions. We recognize that our findings may prompt further investigation into specific mechanisms of action. By presenting a broad analysis, we hope to encourage researchers to delve deeper into individual factors and their interactions, ultimately leading to a more nuanced understanding of how structural racism affects breast cancer outcomes.

## Recommendation and future research direction

Considering the substantial impact of residential segregation and redlining on breast cancer disparities, it is imperative to implement policy interventions that address housing and economic inequities (48). These interventions should focus on dismantling discriminatory lending practices, investing in marginalized communities, and expanding access to affordable housing. Additionally, community-based initiatives, educational programs, and economic empowerment strategies can play a crucial role in alleviating the healthcare barriers that contribute to these disparities.

Within the healthcare system, policy changes are essential for dismantling structural racism. It is critical to ensure equitable access to care, facilitate early detection, and provide high-quality treatment. Furthermore, promoting diversity in the healthcare workforce and implementing culturally competent care practices are essential components of a comprehensive strategy to address these disparities (49).

Future research should aim to enhance our understanding of how structural racism influences breast cancer outcomes, considering the complex interplay of individual and neighborhood factors. Moreover, investigations into other discriminatory social determinants of health (SDOH), including economic and educational disparities, are necessary to elucidate the root causes of breast cancer outcome disparities. This multidimensional approach will be vital for informing effective interventions and advancing health equity in breast cancer care.

## Conclusion

The relationship between structural racism measures and breast cancer outcomes is inherently complex and require comprehensive models. It is crucial to extend the scope beyond current measures, encompassing a diverse range of structural determinants of health such as education, incarceration, and healthcare access. Additionally, there is a pressing need for models that address survivorship experiences, psychological distress, and the quality of care. Moreover, accounting for individual characteristics like race, ethnicity, and comorbidity and considering both small local and broader measures, and understanding their interactions, is essential. Furthermore, studies should delve into moderators of effect and elucidate the mechanisms through which structural measures impact outcomes, both socially and biologically. Lastly, there is a critical need for studies that rigorously test structural racism policy interventions to evaluate their impact on breast cancer outcomes. This comprehensive approach is necessary to address the multifaceted and interconnected factors contributing to disparities in breast cancer outcomes.

## Figures and Tables

**Figure A:**
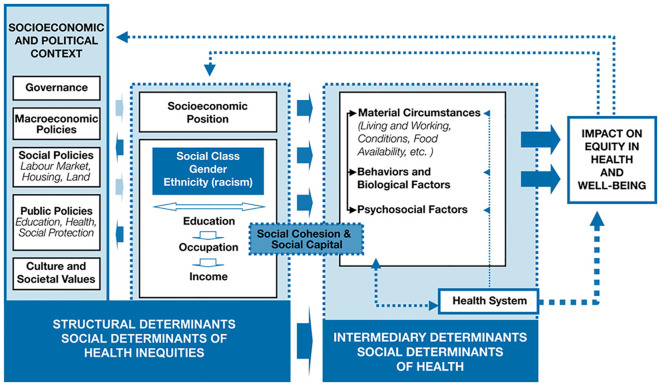
The Commission on Social Determinants of Health (CSDH) World Health Organization (WHO) Framework. The WHO SDOH (Social Determinants of Health) framework highlights structural factors like macroeconomic policies that impact material circumstances and had an impact on health outcomes.

## Data Availability

The original contributions presented in the study are included in the article/supplementary material. Further inquiries can be directed to the corresponding author.

## Appendix

**Summary of Residential segregation measures**

The assessment of residential segregation in these studies employed diverse measures including the five traditional dimensions of residential segregation: evenness, exposure, clustering, concentration, and centralization as well as new small area measures such as Location Quotient measure, the Index of Concentration at Extreme and the local exposure and isolation measures.

Six studies used the Isolation Index which is an **exposure measure** and one used local isolation measure. The isolation index measures the degree of potential contact between groups within neighborhoods of a city. The Isolation Index evaluates the exposure of minority members to either only other minority members or to members of the majority group. Index values range from 0 to 1, with exposure representing the average probability of contact between Blacks and whites at the neighborhood level.

One study used the Delta Index, which is a component of the **Concentration Index**, represents the relative amount of physical space occupied by a minority group in each geographic area. The Concentration Index assesses the population density of Blacks across the metropolitan area relative to the density of white. The index measures the relative amount of physical space occupied by a minority group in the urban environment. The Index of Concentration at Extreme measures the extent to which minority members are exposed exclusively to other minority members or to members of the majority group. Index values range from 0 to 1, indicating the proportion of minority members that would need to move across neighborhoods to achieve a uniform density.

One study used Spatial Proximity which is a **clustering index**. The index measures the extent to which areas inhabited by minority members adjoin one another in space. Spatial Proximity, or clustering, measures the degree to which minority residential areas are adjacent to one another in physical space. Index values range from 1.0 (no difference in clustering) to greater than 1.0 (minority group members living closer to each other than to majority members). Clustering assesses the effect of “ghettoization” or the degree to which predominantly Black neighborhoods are contiguous to predominantly white neighborhoods.

Three studies utilized the Dissimilarity Index, one study employed Theil’s Information Theory Index (H index), and one study utilized Gini, all of which are measures assessing the ‘**evenness**’ of residential segregation. These indices refer to the unequal distribution of social groups across areal units of an urban area. The Racial Dissimilarity Index measures the proportion of minority members that would need to change residence for each unit of analysis to have the same distribution as the larger overall area, ranging from 0 (no segregation) to 1 (complete segregation). Evenness assesses the degree to which each neighborhood mirrors the distribution of Blacks to whites as in the metropolitan area overall. Another measure is the Theil’s Information Theory Index (H index). This index gauges local and regional diversity, evaluating the evenness of racial group distribution across neighborhoods, ranging from 0 (completely even or integrated distribution) to 1 (completely segregated). It approximates the proportion of the minority group that would need to relocate to achieve an even distribution within the region.

One study used Relative **Centralization.** Relative Centralization indicates the proportion of the minority population that would need to change residence to match the centralization level of the majority group. Index values range between −1.0 and 1.0, with positive values indicating that minority group members reside closer to the city center than majority members. The centralization measures the degree to which a group is located near the center of an urban area. Centralization measures how predominantly Black neighborhoods are in relation to the metropolitan area’s center versus its suburbs. Additionally, three studies used the Location Quotient measure.

The Location Quotient (LQ) for racial residential segregation is a comparative measure, assessing the relative proportions of a minority group within a census tract compared to the proportion of that group in the larger Metropolitan Statistical Area (MSA), facilitating the evaluation of disparities in the racial composition of an individual’s neighborhood in relation to the broader MSA.

Redlining measures:

Two measures have been used for redlining: the redlining index and Homeowners’ Loan Corporation (HOLC) grade classification. The redlining index was created by expanding adaptive spatial filters until at least five Black and five white mortgage applicants were identified. Instead of calculating solely within the filter, the odds of mortgage application denial were estimated for individuals inside versus outside the filter. The resulting index, centered around 1, indicates whether an area is equally likely (1), more likely (>1), or less likely (<1) to face mortgage denials compared to other regions in the study area. The original HOLC maps were used to compute the percentage of area in the original maps of each city that were graded A, B, C, or D (corresponding to green = “Best”; blue = “Still Desirable”; yellow = “Declining”; and red = “Hazardous” designations, respectively).

### Table A: Search Strategy-PubMed.

**Table:**

(“built environment”[MeSH Terms] OR “nutrition policy”[MeSH Terms] OR “right to work”[MeSH Terms] OR “zoning policy”[Title/Abstract] OR “redlining”[Title/Abstract] OR “residential segregation”[Title/Abstract] OR “structural inequalities”[Title/Abstract] OR “mass incarceration”[Title/Abstract] OR “unequal educational”[Title/Abstract] OR “educational inequality”[Title/Abstract] OR “wealth accumulation”[Title/Abstract] OR “state sanctioned violence”[Title/Abstract] OR “judicial system”[Title/Abstract] OR “transportation barriers”[Title/Abstract] OR “food access”[Title/Abstract] OR “employment access”[Title/Abstract]) AND (“Breast Neoplasms”[Mesh: NoExp] OR “Carcinoma, Ductal, Breast”[Mesh: NoExp] OR “Hereditary Breast and Ovarian Cancer Syndrome”[Mesh: NoExp] OR “Inflammatory Breast Neoplasms”[Mesh: NoExp] OR “Triple Negative Breast Neoplasms”[Mesh: NoExp] OR ((“breast”[mesh] OR “breast diseases”[mesh:noexp]) AND (“Neoplasms”[mesh:noexp] OR “Adenocarcinoma”[mesh:noexp] OR “Carcinoma”[mesh:noexp])) OR brca[tiab] OR (breast[tiab] AND (adenocarcinoma*[tiab] OR cancer*[tiab] OR carcinoma*[tiab] OR metasta*[tiab] OR neoplasm*[tiab] OR tumor[tiab] OR tumors[tiab] OR tumour[tiab] OR tumours[tiab])))
(“Systemic Racism”[Mesh] OR “Institutional Racism*”[tw] OR “Institutionalized Racism*”[tw] OR “Systematic Racism*”[tw] OR “Systemic Racism*”[tw] OR “Structural Racism*”[tw]) AND (“Breast Neoplasms”[Mesh: NoExp] OR “Carcinoma, Ductal, Breast”[Mesh: NoExp] OR “Hereditary Breast and Ovarian Cancer Syndrome”[Mesh: NoExp] OR “Inflammatory Breast Neoplasms”[Mesh: NoExp] OR “Triple Negative Breast Neoplasms”[Mesh: NoExp] OR ((“breast”[mesh] OR “breast diseases”[mesh:noexp]) AND (“Neoplasms”[mesh:noexp] OR “Adenocarcinoma”[mesh:noexp] OR “Carcinoma”[mesh:noexp])) OR brca[tiab] OR (breast[tiab] AND (adenocarcinoma*[tiab] OR cancer*[tiab] OR carcinoma*[tiab] OR metasta*[tiab] OR neoplasm*[tiab] OR tumor[tiab] OR tumors[tiab] OR tumour[tiab] OR tumours[tiab])))
(“systemic racism”[MeSH Terms] OR (“systemic”[All Fields] AND “racism”[All Fields]) OR “systemic racism”[All Fields] OR (“structural”[All Fields] AND “racism”[All Fields]) OR “structural racism”[All Fields]) AND (“breast neoplasms”[MeSH Terms] OR (“breast”[All Fields] AND “neoplasms”[All Fields]) OR “breast neoplasms”[All Fields] OR (“breast”[All Fields] AND “cancer”[All Fields]) OR “breast cancer”[All Fields])

### Table B: Search Strategy-Embase.

**Table:**

(‘built environment’/exp OR ‘nutrition policy’/exp OR ‘right to work’/exp OR ‘zoning policy’:ti,ab,kw OR ‘redlining’:ti,ab,kw OR ‘residential segregation’:ti,ab,kw OR ‘structural inequalities’:ti,ab,kw OR ‘mass incarceration’:ti,ab,kw OR ‘unequal educational’:ti,ab,kw OR ‘educational inequality’:ti,ab,kw OR ‘wealth accumulation’:ti,ab,kw OR ‘state sanctioned violence’:ti,ab,kw OR ‘judicial system’:ti,ab,kw OR ‘transportation barriers’:ti,ab,kw OR ‘food access’:ti,ab,kw OR ‘employment access’:ti,ab,kw) AND (‘breast tumor’/de OR ‘breast ductal carcinoma’/de OR ‘hereditary breast and ovarian cancer syndrome’/de OR ‘inflammatory breast cancer’/de OR ‘triple negative breast cancer’/de OR ((‘breast’/exp OR ‘breast disease’/de) AND (‘neoplasm’/de OR ‘adenocarcinoma’/de OR ‘carcinoma’/de)) OR ‘brca’:ti,ab,kw OR (‘breast’:ti,ab,kw AND (‘adenocarcinoma*’:ti,ab,kw OR ‘cancer*’:ti,ab,kw OR ‘carcinoma*’:ti,ab,kw OR ‘metasta*’:ti,ab,kw OR ‘neoplasm*’:ti,ab,kw OR ‘tumor’:ti,ab,kw OR ‘tumors’:ti,ab,kw OR ‘tumour’:ti,ab,kw OR ‘tumours’:ti,ab,kw)))
(‘structural racism’/exp OR ‘institutional racism*’:ti,ab,kw,de,dn,df,mn,tn OR ‘institutionalized racism*’:ti,ab,kw,de,dn,df,mn,tn OR ‘systematic racism*’:ti,ab,kw,de,dn,df,mn,tn OR ‘systemic racism*’:ti,ab,kw,de,dn,df,mn,tn OR ‘structural racism*’:ti,ab,kw,de,dn,df,mn,tn) AND (‘breast tumor’/de OR ‘breast ductal carcinoma’/de OR ‘hereditary breast and ovarian cancer syndrome’/de OR ‘inflammatory breast cancer’/de OR ‘triple negative breast cancer’/de OR ((‘breast’/exp OR ‘breast disease’/de) AND (‘neoplasm’/de OR ‘adenocarcinoma’/de OR ‘carcinoma’/de)) OR ‘brca’:ti,ab,kw OR (‘breast’:ti,ab,kw AND (‘adenocarcinoma*’:ti,ab,kw OR ‘cancer*’:ti,ab,kw OR ‘carcinoma*’:ti,ab,kw OR ‘metasta*’:ti,ab,kw OR ‘neoplasm*’:ti,ab,kw OR ‘tumor’:ti,ab,kw OR ‘tumors’:ti,ab,kw OR ‘tumour’:ti,ab,kw OR ‘tumours’:ti,ab,kw)))
(‘structural racism’/exp OR (‘systemic’ AND ‘racism’) OR ‘systemic racism’ OR (‘structural’ AND ‘racism’) OR ‘structural racism’) AND (‘breast tumor’/exp OR (‘breast’ AND ‘neoplasms’) OR ‘breast neoplasms’ OR (‘breast’ AND ‘cancer’) OR ‘breast cancer’)

### Table C: Search Strategy-CINAHL.

**Table:**

(MH “built environment” OR MH “nutrition policy+” OR ((right OR rights) W4 (work* OR employ*)) OR “zoning policy” OR “redlining” OR “residential segregation” OR “structural inequalities” OR “mass incarceration” OR “unequal educational” OR “educational inequality” OR “wealth accumulation” OR “state sanctioned violence” OR “judicial system” OR “transportation barriers” OR “food access” OR “employment access”) AND (MH “Breast Neoplasms+” OR MH “Carcinoma, Ductal, Breast” OR MH “Hereditary Breast and Ovarian Cancer Syndrome” OR “Inflammatory Breast Neoplasm*” OR “Triple Negative Breast Neoplasm*” OR ((MH “breast+” OR MH “breast diseases”) AND (MH “Neoplasms” OR MH “Adenocarcinoma” OR MH “Carcinoma”)) OR brca OR (breast AND (adenocarcinoma* OR cancer* OR carcinoma* OR metasta* OR neoplasm* OR tumor OR tumors OR tumour OR tumours)))
(MH “Systemic Racism” OR “Institutional Racism*” OR “Institutionalized Racism*” OR “Systematic Racism*” OR “Systemic Racism*” OR “Structural Racism*”) AND (MH “Breast Neoplasms” OR MH “Carcinoma, Ductal, Breast” OR MH “Hereditary Breast and Ovarian Cancer Syndrome” OR “Inflammatory Breast Neoplasm*” OR “Triple Negative Breast Neoplasm*” OR ((MH “breast+” OR MH “breast diseases”) AND (MH “Neoplasms” OR MH “Adenocarcinoma” OR MH “Carcinoma”)) OR brca OR (breast AND (adenocarcinoma* OR cancer* OR carcinoma* OR metasta* OR neoplasm* OR tumor OR tumors OR tumour OR tumours)))
(MH “systemic racism” OR (“systemic” AND “racism”) OR “systemic racism” OR (“structural” AND “racism”) OR “structural racism”) AND (MH “breast neoplasms+” OR (“breast” AND “neoplasms”) OR “breast neoplasms” OR (“breast” AND “cancer”) OR “breast cancer”)
